# New directions and opportunities for symmetry-centric structural science

**DOI:** 10.1063/4.0001066

**Published:** 2025-10-27

**Authors:** Branton J. Campbell, Harold T. Stokes

**Affiliations:** Brigham Young University, Provo, Utah, USA

## Abstract

The crystallographic research community has a strong tradition of building and leveraging shared infrastructure for doing high-impact science at the intersection of chemistry, biochemistry, physics, materials science, and geology. Though much has been done already, the opportunities that yet lay ahead are vast and exciting. We will review milestones from our journey in developing symmetry-centric data sources and computational tools for the characterization of phase transitions and structural distortions (publicly available via the ISOTROPY Software Suite) over the past 40 years, and will highlight some of the relatively uncharted frontiers in symmetry-centric structural science that now beckon to new explorers.

Image: Incommensurate 2D skyrmion lattice with magnetic superspace group 177.2.83.6.m153.1 P62'2'(a,a,0)000(-2a,a,0)000.

